# Expanding Horizons in Advancements of FRET Biosensing Technologies

**DOI:** 10.3390/bios15070452

**Published:** 2025-07-14

**Authors:** Munazza Fatima, Naseem Abbas

**Affiliations:** 1 Department of Microbiology, College of Medicine, Gachon University, Incheon 21936, Republic of Korea; munazzafatima@gachon.ac.kr; 2 Lee Gil Ya Cancer and Diabetes Institute, Gachon University, Incheon 21936, Republic of Korea; 3 Department of Mechanical Engineering, Sejong University, Seoul 05006, Republic of Korea

**Keywords:** FRET, cellular imaging, drug discovery, pathogen detection, cancer diagnosis

## Abstract

Förster resonance energy transfer (FRET)-based biosensors are versatile tools for obtaining insights into various biological processes. Their working principles are based on nonradiative energy transfer from donor to acceptor fluorophores. This energy transfer is responsible for a change in fluorescence intensity, which provides a basis for the detection of biomolecules. Advantageous features of FRET biosensors include their high sensitivity and specificity. Recently, there have been notable developments to extend the usage of FRET biosensors for diverse applications. In this review, we briefly summarize the state-of-the-art developments of FRET biosensors for cellular imaging, drug discovery, pathogen detection, and cancer diagnosis. Continued research on biosensor design, donor acceptor pair optimization, and integration of innovative materials can further extend the applications of FRET biosensors across health care settings.

## 1. Introduction

**Figure 1:**
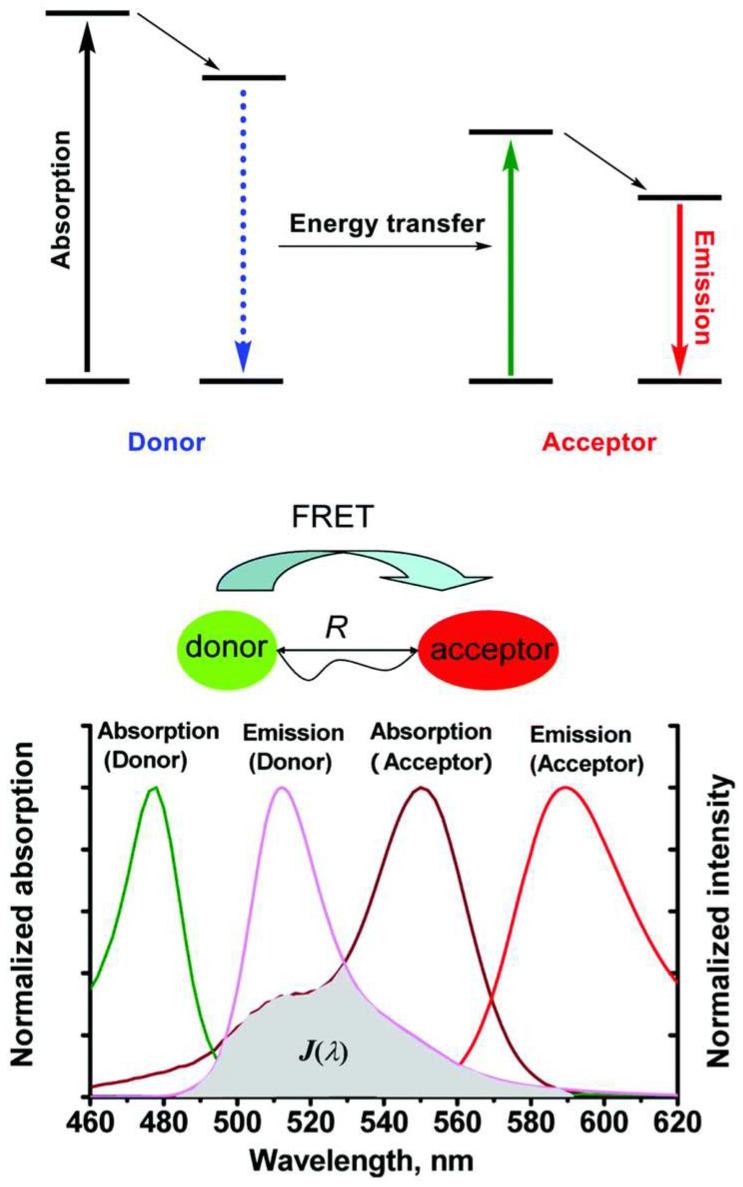
The mechanism of Förster resonance energy transfer (FRET). R represents the distance between the donor and acceptor, while J(λ) (M^−1^cm^−1^nm^4^) represents the degree of spectral overlap between the donor emission and acceptor absorption. Reproduced with permission from reference [5].

Biosensors based on Fluorescence Resonance Energy Transfer (FRET) are powerful diagnostic tools, used to investigate diverse biological processes and pathways at the molecular level [1]. Fluorescent materials are applied as probe molecules in FRET biosensors [2]. Fluorophores, as crucial parts of FRET biosensors, play an important role in energy transfer from donor to acceptor molecules [3]. Their working principle is based on the absorption of light and the excitation of the donor followed by the transfer of energy to acceptors [4]. The mechanism of FRET is shown in Figure 1 [5]. Such energy transfer provides valuable insight into understanding the biological process and the ability to act as a probe to detect biomolecules [6]. The change in fluorescence intensity during the energy transfer in the FRET process is quantitatively linked to the concentration of biomolecules, facilitating its precise detection [7]. The selection of appropriate donor–acceptor pairs of fluorophores is crucial to optimize the performance of biosensors [8]. The distance between the donor and acceptor has a significant influence on the sensor’s ability; in an ideal case, they should be within a few nanometers of each other [9]. The sensitivity of a FRET biosensor is dependent upon the overlapping of donor emission and acceptor absorbance spectra [10]. The adjustment of the relative orientation and the modulation of linkers connecting donor and acceptors can regulate the sensing ability of FRET biosensors [11,12]. The FRET ratio and FRET efficiency are strongly influenced by the photophysical characteristics of fluorophores [13]. The calibration of FRET biosensors is crucial to ensure the reliability of measurements. In the case of imaging sensors, Wu et al. introduced a multiplexed imaging strategy to calibrate biosensors [14] This strategy is useful for the precise measurements of a FRET biosensor for cellular imaging. Structural engineering of biosensors is very important to achieve optimal performance. For instance, the core–shell configuration of a polymer-amplified RNA aptamer Nano Kit has shown highly sensitive and selective performance for the detection of the c-di-GMP biomolecule [5]. Fluorescent proteins-based FRET biosensors usually face the limitations of a small dynamic range because of narrow conformational changes. This shortcoming has been overcome by introducing ER/K linkers into FRET biosensors [6]. This offers a valuable tool for robust cellular imaging. Although versatile strategies have been adopted to enhance the sensitivity and specificity of FRET biosensors, there is still room for further improvements. The efficient sensing ability of FRET biosensors can be greatly enhanced by tuning the composition of fluorophores and optimizing the phenomena of intermolecular energy transfer. Similarly, integration of advanced nanomaterial such as up-converting nanoparticles and conjugated polymers can greatly boost the performance of FRET biosensors [15,16]. Moreover, it is challenging to control the photobleaching- and environmental factors-dependent decrease in the fluorescence intensity and FRET signals [17]. Overcoming such obstacles is essential to fully exploit FRET biosensors for diverse applications. The following section describes the working principle and state-of-the-art developments in the design of efficient FRET biosensors for a range of applications including cellular imaging [18], drug discovery [19], pathogen detection [20], and cancer research [21].

### Principle of FRET

FRET governs nonradiative energy transfer from an excited donor under illumination to an acceptor [22]. Light absorption by molecules causes excitation from the ground state. The excited molecules can be relaxed in different modes. In simple florescence phenomena, excited states can undergo emission of a photon to reach the ground state in a radiative pathway. However, in the FRET process, a donor can transfer energy to a nearby acceptor fluorophore in a nonradiative energy transfer without emitting a photon, as shown in Figure 1 [5].

Columbic interaction affects FRET, reflecting that energy transfer between donor and acceptor fluorescent molecules occurs through dipole–dipole interactions [23]. The phenomena of such energy transfer are highly influenced by the distance between the acceptor and donor [24]. If, however, the distance between them is within 1–10 nanometers, the energy transfer process can be effectively executed [22]. It can be anticipated that if there is a larger spectral overlap between donor and acceptor or there is a shorter distance between donor and acceptor, the energy transfer efficiency could be higher [25]. The FRET efficiency can be calculated from the following equation, where $R_{0}$ defines the Förster radius, i.e., the distance at which the energy transfer efficiency is 50%, whereas R denotes the donor–acceptor pair distance [26].

(1) $$\;E_{FRET}=\frac{R_{0}^{6}}{R_{0}^{6}+R^{6}\;}$$

However, if there is a single donor and multiple (*n*) acceptors, then the FRET efficiency can be expressed as follows [27].

(2) $$E_{FRET}=\frac{{nR}_{0}^{6}}{{nR}_{0}^{6}+R^{6}\;}$$

The $R_{0}$ is an important parameter affecting the FRET efficiency, and the theoretical expression adopted to calculate $R_{0}$ is as follows [28].

(3) $$R_{0}=\left(\frac{Q_{D}\;J(\lambda )[9000\;(\ln10)]\;K^{2}}{128\;\pi^{5}\;n^{4}\;N_{A}}\;\right)\cdot^{1/6}$$

In the above expression, $Q_{D}$ represents the quantum yield, *J*(λ) the spectral overlap, $K^{2}$ the relative orientation factor, *n* the refractive index of medium, and $N_{A}$ the Avogadro number. Whereas the *J*(λ) can be obtained from the following expression. In this expression, $F_{D}$ denotes the donor emission profile, and ℇλ is the acceptor molar extinction coefficient [29].

(4) $$J\left(\lambda \right)=\int_{0}^{\infty }F_{D}\left(\lambda \right)ℇ\lambda^{4}\;d\lambda$$

The concept of FRET is commonly applied in diverse applications related to biological systems to obtain insights into various biological phenomena, as well as for the detection of biological molecules and diseases [30]. It promotes the fundamental understanding of biological systems at a molecular level. The following section provides a detailed discussion on extending the FRET application into various fields.

Unlike FRET biosensors for bulk measurements, single-molecule FRET (smFRET) provides unique advantages in term of sensing resonance energy transfer at a molecular level. In this case, a single donor and acceptor are involved in the execution of the FRET response. Some of the conformational changes are not easily detected by using bulk FRET sensors, which could be made possible with the help of smFRET, enabling it to be a powerful tool at a molecular scale. Notable advancements have been made in probing the conformational changes in protein and nucleic acid regulation [31].

## 2. Applications for FRET Biosensors

**Figure 2:**
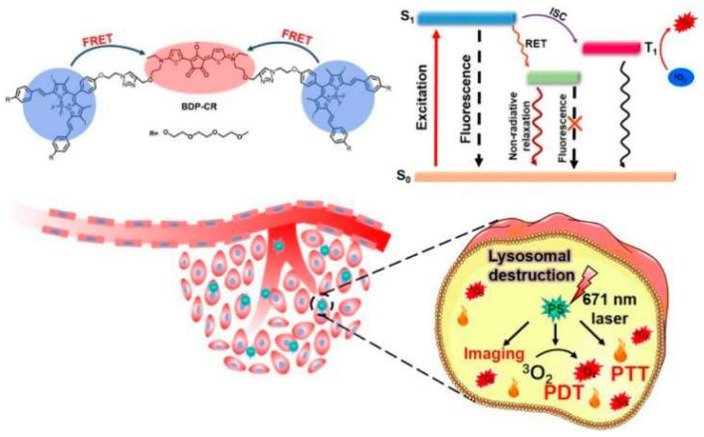
Schematic illustration of the smFRET-based combination phototherapy mechanism (BDP-CR) as well as light-triggered cancer cell death. Reproduced with permission from reference [33].

FRET biosensors have found widespread applications across various fields of biomedical science, due to their ability to detect molecular interactions with high sensitivity and spatial–temporal resolution. In the context of cellular imaging, they allow real-time monitoring of intracellular events such as protein–protein interactions, enzyme activities, and ion concentration changes. Their utility in drug discovery lies in their capacity to evaluate molecular responses to candidate drugs, making them suitable for high-throughput screening platforms. For pathogen detection, FRET biosensors offer rapid, specific, and sensitive identification of infectious agents by targeting nucleic acids or proteins unique to pathogens. In cancer diagnosis, they contribute to early-stage detection by sensing tumor-specific biomarkers and alterations in cellular signaling pathways. These diverse applications demonstrate the adaptability and impact of FRET biosensors in both research and clinical settings. Genetically encoded tension sensors (GETS) are usually composed of FRET pairs. Liu et al. demonstrated the development of digital GETS for precisely measuring the tension in a living system [32]. Zou et al. applied the concept of smFRET as a photosensitizer to achieve the combined effect of photodynamic therapy (PDT) and photothermal therapy (PTT) [33]. Such a synergetic effect has been found useful to kill cancer cells in vivo and vitro. Light absorption characteristics have been improved in the NIR region, where excitation at 671 nm has resulted in the generation of reactive oxygen species (ROS) as well as the PTT effect, as shown in Figure 2. One experiment utilized a BODIPY-chlorin (BDP-CR) photosensitizer system to achieve the theranostic effects, where BDP-CR molecules, linked by a PEG chain, enabled fluorescence resonance energy transfer (FRET) between blue and red fluorophores, initially quenching the fluorescence. In the presence of a 671 nm laser, the system transitioned to an excited state (S_1_), facilitating intersystem crossing (ISC) and resonance energy transfer (RET) to ROS, while the nonradiative relaxation was minimized. This process was applied to lysosomes within cells, as shown in the cellular image, where laser-induced ROS production leads to lysosomal destruction, PDT, and PTT, with imaging capabilities enhanced by ^3^O_2_ generation, enabling simultaneous diagnosis and treatment.

**Figure 3:**
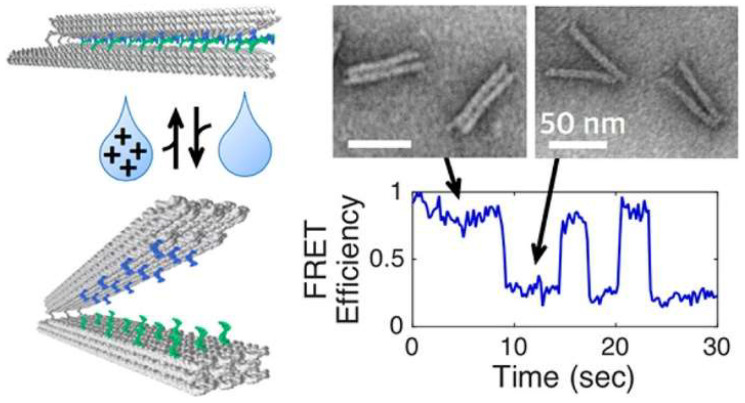
Ionic-dependent structural reconfiguration of DNG hinges probed by smFRET. Reproduced with permission from reference [34].

Marras et al. fabricated a DNA-based origami device, which works based on the change in ionic conditions [34]. It enabled structural reconfiguration in the form of closing and opening DNA hinges on a millisecond time scale, observed based on the sensing behavior of smFRET, as shown in Figure 3. However, aside from its advantages, smFRET also experiences some drawbacks including limitations for multidimensional confirmation analysis of proteins, as well as facing immobilization challenges at a molecular scale.

### 2.1. Cellular Imaging

FRET biosensors are frequently applied for cellular imaging, providing insight into biological processes. Taking advantage of nonradiative energy transfer, FRET biosensors can determine the changes in living cells. These are helpful to understand the interactions at the molecular level. This has resulted in the wider application of FRET biosensors for cellular imaging, providing the advantages of superior resolution. Notable developments of FRET biosensors in the field of cellular imaging are outlined in the following section.

#### 2.1.1. Disease-Targeted Cellular Imaging Using FRET Biosensors

**Figure 4:**
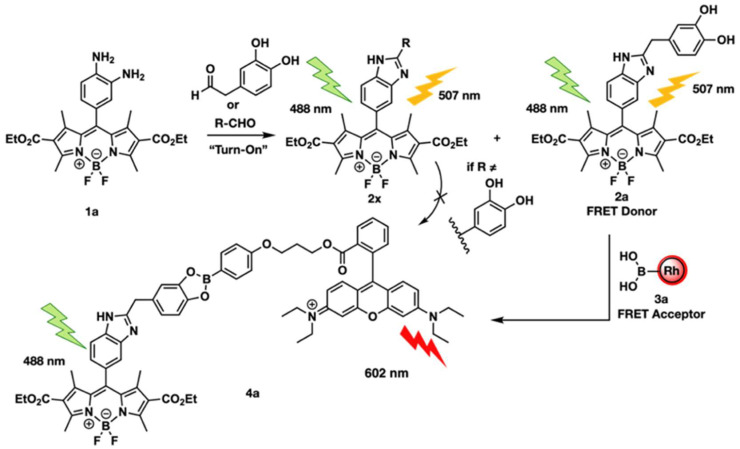
“Turn-On” fluorescence of probe 1a with aldehydes and selective FRET formation of the catechol benzimidazole FRET donor 2a, which reacts with the boronic acid FRET acceptor 3a to create the detectable FRET pair 4a. Other aldehyde products (2×) cannot form a FRET pair. Reproduced with permission from reference [35].

Talbott et al. integrated FRET with fluorescence lifetime imaging (FLIM) for detecting catechol aldehydes (CAs) in neural tissues [35] Their developed sensor is promising in detecting neurodegenerative diseases without potential interference from other biological substances. In their design, diamine-phenyl-BODIPY 1a reacts with aldehyde to produce benzimidazole-BODIPY 2a. Through catechol, it reacts with phenyl boronic acid-functionalized Rhod-amine B 3a to generate RhoB-boronic ester 4a, as shown in Figure 4. 2a acts as FRET donor, while 3a acts as a FRET acceptor. This designed probe demonstrated the efficient detection of CAs in living cells.

**Figure 5:**
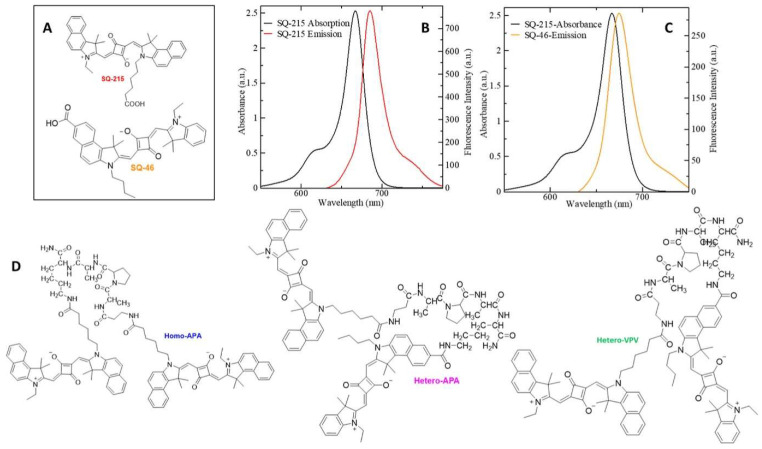
Structure and photophysical characterization of Homo-APA: (**A**) structure of dyes SQ-215 and SQ-46 (fluorophores), (**B**) overlap of absorption and emission spectra of 10 μM SQ-215 in CHCl_3_, (**C**) overlap of absorption and emission spectra of 10 μM of SQ-215 and SQ-46 in CHCl_3_, and (**D**) structures of the FRET-based probes. Reproduced with permission from reference [36].

**Figure 6:**
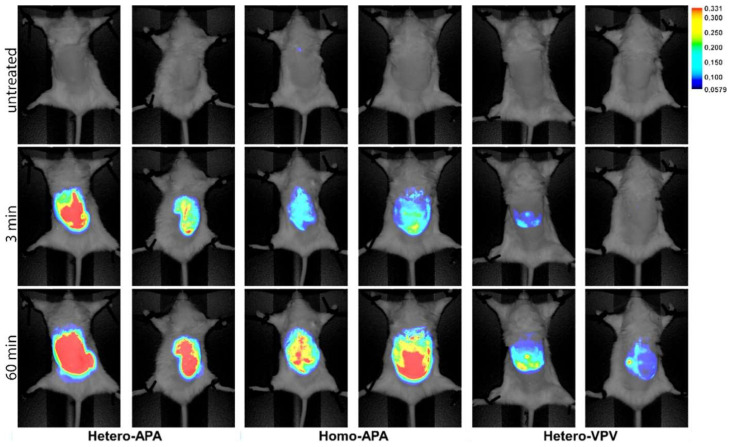
In vivo comparison of FRET-based probes in the air pouch with MSU-induced NETs. Reproduced with permission from reference [36].

Mavileti et al. used a FRET biosensor for the detection of neutrophil extracellular traps (NETs). The authors developed a peptide-based FRET biosensor for the detection of NETosis by investigating the protease activity of neutrophil elastase (NE) [36]. It resulted in the efficient detection of the NETs. For fabricating the FRET biosensor, dyes SQ-215 and SQ-46 were integrated with Ala-Pro-Ala (APA) and Val-Pro-Val (VPV) substrates. The structures of the probes and their integration with APA and VPV in homo and hetero configurations are shown in Figure 5. Figure 5B,C show that hetero configuration has better spectral overlap between the absorption and emission. It has translated into an improvement in the efficiency of the FRET probe for detecting NETs, induced by injecting monosodium urate (MSU) crystals in mice, as shown in Figure 6.

Figure 6 shows the in vivo fluorescence imaging of mice to evaluate the biodistribution and targeting efficiency of squaraine-peptide conjugates: Hetero-APA, Homo-APA, and Hetero-VPV. The mice were intravenously injected with each conjugate, and the fluorescence images were captured at three points: untreated (baseline), 3 min post-injection, and 60 min post-injection. The images display the fluorescence intensity overlaid on grayscale anatomical images, with a heatmap scale indicating the signal strength (from blue to red). At 3 min, all three probes showed some accumulation in the abdominal region, but Hetero-APA and Homo-APA demonstrated stronger and more widespread signals than Hetero-VPV. At 60 min, both APA conjugates showed persistent and intense signals, indicating greater tissue retention or targeting efficiency, while Hetero-VPV exhibited comparatively weaker fluorescence. This experiment highlights the differential in vivo performance of structurally distinct squaraine-peptide probes and underscores the influence of the peptide composition on the probe accumulation and retention in live subjects.

#### 2.1.2. Enhancing the Signal Stability and Imaging Quality in Live Cells

**Figure 7:**
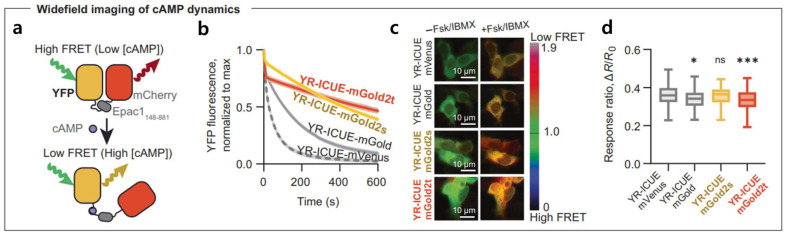
(**a**) Schematics of YR-ICUE, a FRET-based cAMP indicator. FRET fluorescence resonance energy transfer, cAMP cyclic adenosine monophosphate, Epac1 exchange protein directly activated by cAMP 1. (**b**) YR-ICUE variants with mGold2s and mGold2t showed enhanced photostability under widefield imaging compared with mVenus or mGold. Excitation: 520-nm light at 17 mW/mm^2^. Mean ± 95% CI, *n* = 6 independent transfections. (**c**) Representative pseudocolor images showing YR-ICUE responses before and after adding Fsk and IBMX. Fsk forskolin, IBMX 3-isobutyl-1- methylxanthine. (**d**) Fluorescence responses were comparable for YR-ICUE-mGold2s and YR-ICUE-mVenus, while YR-ICUE-mGold2t showed a slightly diminished response. R represents the RFP/YFP emission ratio. R0 is the emission ratio immediately preceding drug addition. Boxplots of 153–225 cells are shown. Center lines, median; box limits, upper and lower quartiles; whiskers, 1.5× interquartile range. * *p* = 0.0174; *** *p* = 0.0008; ns, *p* > 0.05; Dunnett’s T3 multiple comparisons test after two-sided Welch’s ANOVA test. Reproduced with permission from reference [37].

Photobleaching of fluorescent proteins is a major drawback, responsible for decreasing FRET efficiency. In order to overcome this issue, Lee et al. demonstrated a significant improvement in the photostability of yellow fluorescent proteins (YFPs) [37]. In their proposed system, they integrated adenosine monophosphate (cAMP) indicator YR-ICUE with mGold, mGold2s, and mGold2t. YFPs and mCherry act as the FRET donor and acceptor, respectively, as shown in Figure 7a. Compared to YR-ICUE-mVenus, YR-ICUE-mGold2s and YR-ICUE-mGold2t show a significant improvement in resisting photobleaching, as shown in Figure 7b. This resulted in a higher FRET response and bright imaging, as shown in Figure 7c; in Figure 7d, the fluorescence responses were comparable for YR-ICUE-mGold2s and YR-ICUE-mVenus, while YR-ICUE-mGold2t showed a slightly diminished response. R represents the RFP/YFP emission ratio.

**Figure 8:**
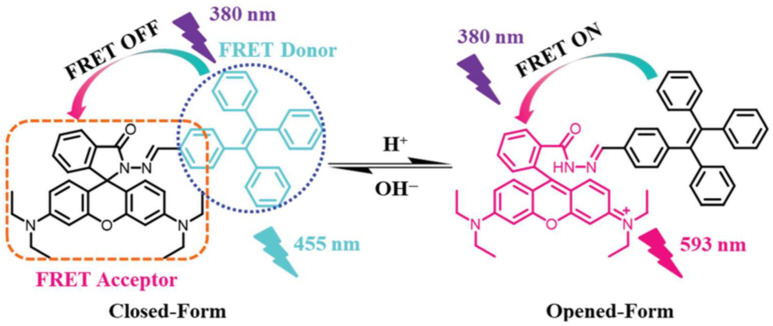
Structure design and the corresponding response mechanism of Rh-TPE towards pH through the process of FRET. Reproduced with permission from reference [42].

In addition, FRET biosensors were also adopted to investigate the protein–protein interactions in live cells, which provides useful information to understand the cell signaling pathways. For instance, Kagan et al. developed a FRET biosensor based on Phosphatase and Tensin Homolog (PTEN) confirmations to track the PTEN activity, which allowed the dynamic imaging of PTEN activity in cell lines [38]. The PTEN activity in this work was co-related with two photon fluorescence lifetime imaging spectroscopies. This finding provides a reliable approach to investigate PTEN activity in living cells. It is important to consider that FRET biosensors can detail the molecular level interactions, which are difficult to observe using conventional biochemical investigations, providing a better understanding at the cellular level [39]. Genetically encoded FRET biosensors are useful for imaging analysis for probing metabolites at the cellular level. These can be supportive in analyzing molecular level interactions and cellular metabolism, using the fluorescence lifetime imaging microscopy (FLIM) approach [40]. The capabilities of FRET biosensors in cellular imaging can be regulated by modifying certain parameters such as the pH and ionic concentrations’ effect [41]. Maintaining a suitable pH of the cells is very important to maintain their good functioning. Probing the cellular pH is an important aspect to monitor the physiological process. FRET biosensors provide a versatile tool for monitoring cellular pH conditions [41]. Chen et al. [42] demonstrated the development of a rhodamine-based (Rh) sensor. In this design, tetraphenylethylene (TPE) acts as a donor, while Rh acts as an acceptor. The pH dependent response in the FRET energy transfer mechanism of the Rh-TPE system is highlighted in Figure 8. A linear response of the Rh-TPE biosensor is observed in the pH range of 3.3–5.0. It shows the suitability of the designed pH sensor for biological applications [42].

#### 2.1.3. FRET Biosensor for Monitoring Mitochondrial Autophagy

**Figure 9:**
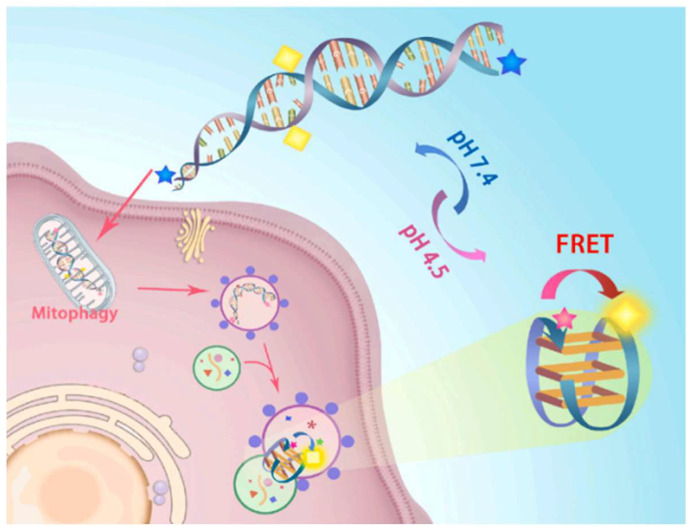
Schematic diagram of mitochondrial autophagy and recognition mechanism of P24-mid. Reproduced with permission from reference [43].

In the context of mitochondrial autophagy (mitophagy), FRET biosensors can also detect molecular changes related to mitochondrial degradation and recycling within cells. By employing donor and acceptor fluorophores linked to specific mitochondrial or autophagy-related molecules, these biosensors enable real-time visualization and quantification of mitophagy events. This approach provides valuable insights into mitochondrial health and dysfunction, which are critical in various diseases and cellular stress responses. Yang et al. demonstrated the construction of a pH responsive G-quadruplex DNA-based FRET biosensor for investigating mitochondrial autophagy [43]. In this work, a biosensor was constructed using oligonucleotide P24, which transforms into a double-stranded structure at the mitochondrial pH. The P24 was integrated with Cy3 and Cy5 fluorophores to design a biosensor for the real-time monitoring of mitochondria. The pH responsive functioning of the FRET biosensor for mitophagy application is shown in Figure 9.

This figure illustrates a pH-sensitive FRET-based DNA nanodevice designed for monitoring mitophagy in living cells. The DNA probe is labeled with a donor (blue star) and acceptor (yellow square) fluorophore pair, enabling FRET in response to environmental pH changes. Under physiological pH (7.4), the fluorophores remain apart, resulting in low FRET efficiency, whereas, in acidic conditions (pH 4.5), such as those found in lysosomes, the DNA structure undergoes a conformational change that brings the donor and acceptor into close proximity, triggering a strong FRET signal. Upon cellular uptake, the probe is trafficked through the endocytic pathway and accumulates in autolysosomes during mitophagy, where the acidic microenvironment activates FRET, allowing real-time visualization of mitophagy progression through fluorescence microscopy.

#### 2.1.4. FRET Biosensor for Cardiac and Kinase Activity

FRET biosensors have been extended to cardiac ryanodine pathological applications. A biosensor based on donor-FKBP12.6 and acceptor-CaM proved its ability to investigate the pathological leaky state of the cardiac ryanodine receptor (RyR2). It has been useful to interpret the regulation of calcium leak in cardiomyocytes [44]. Ouyang et al. [45] demonstrated the design of a FRET biosensor for probing C-terminal Src kinase (CSK) activity in the cells. A FRET biosensor was developed to visualize CSK activity in live cells. The designed sensor has been useful to detect CSK activity and its existence in lipid and non-lipid membrane microregions, confirming its importance for understanding cellular processes [45]. In this design, enhanced cyan fluorescent protein (ECFP) and a variant of yellow fluorescent protein (YPet) played the role of FRET donor and acceptor, respectively. The tyrosine-phosphorylated and tyrosine-dephosphorylated modes greatly influence the FRET efficiency, as well as the CSK kinase activity. In the unphosphorylated mode, ECFP and YPet are closer to each other, resulting in a higher FRET efficiency. In tyrosine-phosphorylated configuration, the kinase binding to the SH2 pocket may cause a distance between the YPet and ECFP, which causes a decrease in the FRET efficiency. Although, there has been considerable in FRET-based biosensors for visualizing the cellular scale, there are still limitations of poor sensitivity, which limit their further application. Overcoming such shortcomings can further boost the application of FRET biosensors for biological applications. Ouyang et al. [46] designed CFP/YPet to improve the sensitivity of a FRET biosensor. This resulted in a several-fold improvement in the sensitivity of a FRET biosensor for detection various molecules such as rosine kinase Src, small GTPase Rac, calcium, and a membrane-bound matrix metalloproteinase MT1-MMP. Such an improvement in sensitivity can boost the understanding of cellular phenomena [46]. FRET biosensors provide useful information to monitor biological phenomena in living tissues, including the liver. Tao et al. [13] demonstrated the application of a FRET biosensor with living tissues to monitor mice livers. They integrated FRET phenomena with two-photon excitation (2PE) microscopy. The fluorescent proteins of mTurquoise and mVenus demonstrated higher spatiotemporal resolutions in imaging the live cells [13].

### 2.2. Drug Discovery

FRET biosensors have emerged as a useful tool in drug discovery, due to their sensitivity and ability to monitor dynamic interactions at molecular levels. These facilitate tracking changes that occur at a cellular scale without having a negative influence on cellular processes. It allows extending their applications to investigate enzyme inhibition, protein–protein interactions, conformational changes, and determination of the mechanisms. Such features enable FRET biosensors to be an important tool in diagnosis and drug discovery. FRET and time-resolved FRET are frequently applied for drug validation. This provides insight into drug efficacy and mechanism. Such valuable information is helpful to design advanced therapeutics [47]. Innovative approaches involving FRET biosensors for drug discovery are highlighted as follows. FRET biosensors have been applied for the real-time detection of NADPH in cells, after exposure to drugs. It has been helpful to unfold the impact of drugs on free NADPH in living cells [48]. Roopnarine et al. [49] demonstrated the application of a FRET biosensor to probe the phenomena of cardiac sarco/endoplasmic reticulum calcium ATPase (SERCA2a). Their work demonstrated the importance of FRET biosensors to investigate the activators and inhibitors involving cardiac therapeutics [49]. Muretta et al. [50] explored TR-FRET to sense the allosteric modulators based on changes in protein structure. The donor and acceptor pair were composed of Alexa-488 and Cy3-ATP/ADP. This provided useful insight for sensing cardiac myosin. This finding reflects the significance of allosteric modulators in drug discovery [50]. Schultz et al. [51] investigated drugs that facilitate the formation of the SERCA2a/DWORF complex. They investigated the correlation of FRET with Ca-ATPase activity, which could be useful to develop a drug for heart disease [51]. Alpha-synuclein (aSyn) protein is corelated with Parkinson’s disease. Aggregation of aSyn could be responsible for causing Parkinson’s disease. Yu et al. [52] in a recent finding, explored the early detection of aSyn aggregation based on applying FRET technology. Their finding showed that Ser129 phosphorylation (pS129) promoted α-Syn aggregation. In this FRET design, CY3 and CY5 worked as donor and acceptor, respectively [52]. Braun et al. [53] upgraded an aSyn FRET biosensor. The newly designed sensor minimized the non-specific FRET response and significantly enhanced transient transfection. It has been helpful in reducing the cellular toxicity induced by overly expressed aSyn. This finding could facilitate the development of neurodegeneration therapeutics [53].

#### 2.2.1. FcRn Antagonist Screening Using Phase Transition-Based FRET (PT-FRET)

**Figure 10:**
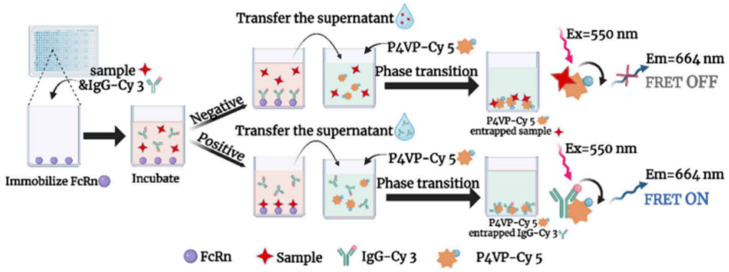
Schematic illustration of the PT-FRET technique. Reproduced with permission from reference [54].

Neonatal Fc receptor (FcRn) antagonists are applied for IgG-mediated autoimmune disorders. Wu demonstrated the application of phase transition-based FRET (PT-FRET) to screen out potential FcRn antagonists [54]. It was integrated with pH-responsive polymers, which greatly amplified the fluorescence response. Virtual screening was also performed to determine the interaction of FcRn. FcRn antagonists are usually composed of macromolecules; however, this finding demonstrated a shift from macromolecules to small molecules. In this configuration, Cyanine 3 (Cy 3) and Cyanine 5 (Cy 5) acted as donor and acceptor. Cyanine 3-labeled IgG were introduced for binding to FcRn, whereas Cy 5, labeled P4VP, were later introduced. A pH-dependent phase change occurs to P4VP, which is related to the FRET response. Binding small molecules with FcRn antagonism will exhibit FRET phenomena, whereas the absence of FRET reflects the nonexistence of FcRn antagonism. The working principle of PT-FRET is highlighted in Figure 10. This approach has extended the application of FRET to drug screening for FcRn antagonism.

#### 2.2.2. FRET-Based Drug Efficacy Screening in Cancer Models

Funato et al. [55] demonstrated the application of homogeneous time-resolved FRET (HTR-FRET, HTRF) to investigate interactions among phosphatase of regenerating liver (PRL) and cyclin M (CNNM) membrane proteins. This approach was applied to screen the compounds effective for inhibiting PRL-CNNM interactions. Such findings are helpful for drug discovery for cancer treatment [55].

**Figure 11:**
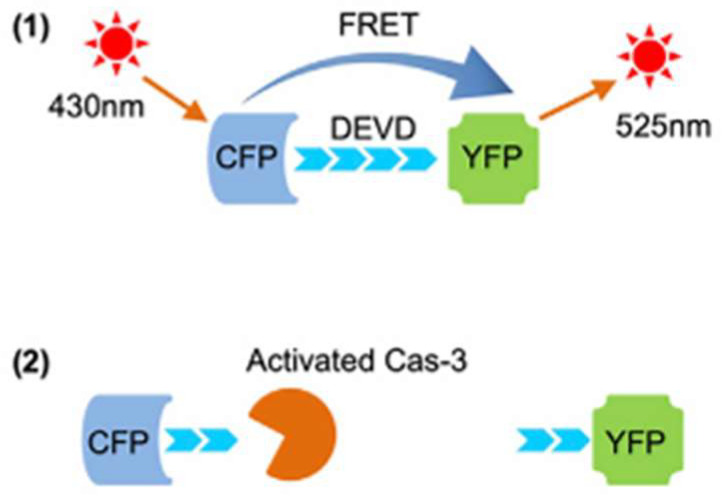
Principle of caspase-3 reporter Sensor C3. Reproduced with permission from reference [56]. Principle of caspase-3 reporter Sensor C3, (1) ransfer energy from CFP to YFP, causing the emission of green fluorescence, (2) real-time apoptosis analysis.

Xing et al. explored the application of FRET to screen drug efficiency in zebra fish and mice models for in vivo studies [56]. They demonstrated the transfection of cancer cells with a FRET sensor to evaluate the cell apoptosis caused by the drug. The outcome of this finding has been useful to detect the death of cancer cells due to drug treatment by mapping the change in the FRET signal. The working principle of the FRET sensor adopted in this work is shown in Figure 11. FCP and YFP function as the FRET donor and acceptor, respectively. During cell apoptosis the FRET energy transfer from CFP to YFP is reduced, which is used to determine apoptosis.

**Figure 12:**
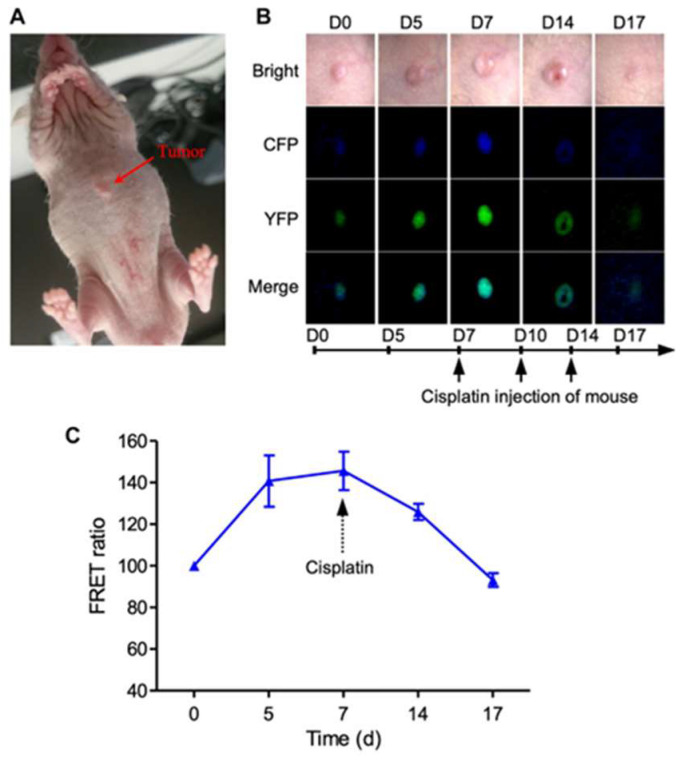
Imaging of cell apoptosis in xenograft tumor mouse in vivo. (**A**) Imaging of 231-C3 xenograft nude mice and FRET channels of a tumor with no drug treatment. Here, 100 µL 5 × 10^6^/mL 231-C3 cells were injected into each site of nude mice. Mice were injected with 6 mg/kg cisplatin after the establishment of the tumor xenograft. (**B**) Imaging of 231-C3 xenograft tumor nude mice with cisplatin treatment. (**C**) Quantification of FRET ratio in xenograft nude mice tumor after 6 mg/kg cisplatin treatment. In vivo imaging of mouse tumor was performed using a Leica Leica M165FC Fluorescent Stereo Microscope (Wetzlar, Germany). CFP (ex: 440 nm; em: 480 nm), YFP (ex: 440 nm; em: 535 nm). Data are expressed as mean ± s.e.m. *n* = 3. Reproduced with permission from reference [56].

The imaging of cell apoptosis in mice and the effect of injecting cisplatin into mice is shown in Figure 12. This reflects the effectiveness of the designed platform for screening anticancer drugs.

The experiment involved a mouse model with a tumor, as shown in panel A, where a tumor is marked on the mouse’s back. Panels B and C provide the detailed observations over time following a cisplatin injection. Panel B displays the fluorescence imaging with CFP and YFP channels, merged to show colocalization, tracked from day 0 (D0) to day 17 (D17), indicating changes in tumor fluorescence over the treatment period. Panel C presents a graph of the FRET ratio over time (days 0 to 17), showing an initial increase peaking around day 5, followed by a decline after the cisplatin injection, suggesting a dynamic response in the tumor’s molecular interactions, likely reflecting the drug’s effect on the tumor cells.

Additionally, FRET technology has been used for the earlier detection of extracellular vehicles (EVs) causing Alzheimer’s disease. Gu et al. [57] demonstrated the EV proteins of amyloid-β (Aβ42) and CD63 by applying a FRET biosensor composed of Au nanoclusters and polydopamine. This provides an effective strategy for determination of the early-stage development of Alzheimer’s disease [57]. The FRET biosensors were applied to investigate protein integration and aggregations; understanding their pathway and precise detection could help to prevent disease progression. TAR DNA-binding protein 43 (TDP-43) exists in Alzheimer’s, ALS, and FTD diseases. Kochen et al. applied fluorescence lifetime-based FRET biosensors to investigate TDP-43 interactions with drugs [58]. This provides a valuable platform for understanding the interactions of TDP-43 for therapeutic developments. Kim et al. [59] developed a single molecule FRET to probe the mechanistic aspects and drug screening for type II topoisomerases. This has been useful to analyze the interaction of a drug with a protein, facilitating the drug screening process [59]. Estrogen receptors (ERs) play an important role in regulating cellular growth. Han et al., [60] in a recent finding, explained the potential of FRET biosensors in monitoring the dimerization and translocation of ERs. This provides a basis for screening the drugs, facilitating ER dimerization [60]. The above findings have revealed encouraging results for extending the application of FRET toward drug discovery. However, despite the ability of FRET sensors to monitor molecular interactions, there are existing barriers that limit their potential in this field. These include interference in complex biological environments and inefficient delivery to the living cells. This could be greatly improved by optimizing the FRET composition and carefully choosing the linker design. Furthermore, the integration of emerging technologies such as artificial intelligence could be useful to exploit the role of FRET in drug discovery and therapeutic applications [41].

### 2.3. FRET Biosensors for Pathogen Detection

FRET biosensors are emerging as an important diagnostic tool for detecting pathogens. These have the advantages of higher sensitivity and facile and rapid analysis, as compared to conventional detection methods. The concept of nonradiative energy transfer between fluorophores is used for the detection of pathogens in various fields including clinical settings, as well as food born illnesses and environmental monitoring. Integrating FRET biosensors for the detection of various pathogens along with their comparative advantages are described in the following sections.

#### 2.3.1. Detection of Bacterial Pathogens

**Figure 13:**
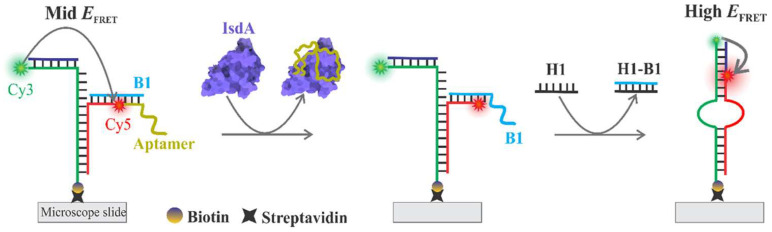
Design for the detection of IsdA. The DNA construct is composed of five different oligonucleotides, two of which are labeled with a Cy3 or a Cy5 fluorophore. The construct assumes a mid-FRET state but switches to a high-FRET state when IsdA pulls out the aptamer upon binding to it, thereby letting the H1 strand bind and displace the B1 strand and ultimately switching the construct to a high-FRET state (detection). Reproduced with permission from reference [61].

Wijesinghe et al. demonstrated the application of a FRET biosensor to detect *Staphylococcus aureus* (*S. aureus*) bacteria, which is a commonly existing pathogen in foods [61]. This was based on the detection of the protein iron-regulated surface determinant protein A (IsdA). This work highlights the significance of FRET technology for the early-stage and precise detection of pathogens in the food industry. Using this approach, the highly sensitive detection of IsdA has been realized in the range of picomolar levels (×10^−12^ M, equivalent to ~1.1 femtomoles IsdA), having a dynamic range of ~40 nM. This has been made possible by the integration of FRET with RNA aptamers to realize the detection at the single molecular scale. The detection of IsdA is highlighted in Figure 13 [61]. In addition, Figure 13 depicts a FRET assay on a microscope slide, where a DNA aptamer labeled with Cy5 and Cy3 fluorophores is anchored via biotin–streptavidin binding. Initially, the aptamer exhibits mid-level FRET efficiency as it interacts with the IsdA protein, causing a conformational change. This is followed by the addition of hairpin DNA (H1), which binds to the aptamer-IsdA complex, increasing the FRET efficiency to a moderate level, due to proximity changes. Subsequently, the addition of H1-B1 further enhances the FRET efficiency to a high level, as the system stabilizes, with the final structure showing strong energy transfer between Cy3 and Cy5, indicating successful molecular recognition and binding events.

**Figure 14:**
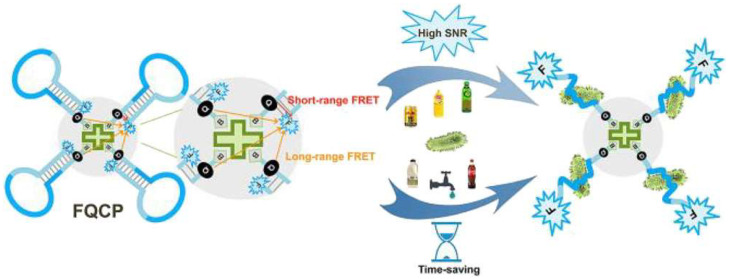
AEMB-based formation of FQCP with the short- and long-range FRET effects. Reproduced with permission from reference [62].

Salmonella is another foodborne pathogen that could be responsible for certain diseases. Zhang et al. developed a FRET-based probe for the detection of Salmonella, which provides the advantages of an easy method and precise results compared to the conventional approaches adopted for its detection [62]. This is based on designing a fluorescence-quenching collapsar probe (FQCP) using an aptamer-encoded molecular beacon (AEMB). The FQCP has been integrated with short- and long-range FRET to minimize the background noise and enhancing the rapid detection of Salmonella within 30 min. Figure 14 shows that the FQCP has four binding positions for Salmonella, which can significantly enhance its detection ability. The designed probe has been found useful for the precise detection of Salmonella in various beverages including water, milk, tea, Coco Cola, juices, etc. Furthermore, it has been observed that the designed system reflected high specificity for Salmonella and negligible fluorescence intensity for the non-target pathogens. The experiment utilizes a Fluorescence Quencher and Crosslinker Platform (FQCP) featuring a central crosslinker with four DNA arms, designed to detect multiple targets through FRET. Initially, the system employs long-range FRET and short-range FRET to detect targets, with the crosslinker facilitating energy transfer between the fluorophores and quenchers. Upon target binding (e.g., bacteria, viruses, or chemicals like bottles and faucets), the system transitions to a high signal-to-noise ratio (SNR) state, indicated by enhanced fluorescence signals, which are achieved through time-saving optimization. This setup allows for the efficient and sensitive detection of various analytes by leveraging the spatial arrangement and energy transfer dynamics within the FQCP.

**Figure 15:**
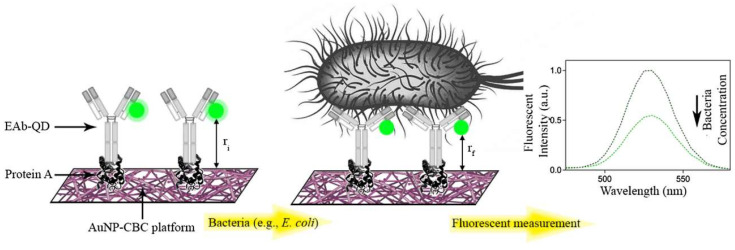
The operational concept of the proposed immunosensor. The carboxylated bacterial cellulose impregnated with AuNP (CBC-AuNP) is implemented as a solid platform to support the essential biorecognition elements, protein A, and labeled antibody with fluorophores. Upon recognizing bacteria, a conformational change occurs in the 3D structure of the EAb-QD that leads to the decrease in the QD photoluminescence by the immunosensor. This phenomenon is due to the reduction in the effective distance between the donor (QD) and acceptor (AuNP-CBC) from r_i_ to r_f_. Reproduced with permission from reference [63].

Heli et al. demonstrated a plasmonic-based FRET immunosensor to detect *Escherichia coli* (*E. coli*) bacteria [63]. This platform was developed on paper, wherein, carboxylate bacterial cellulose (CBC) and gold nanoparticles (AuNP-CBC) were designed for protein conjugation. Bacterial detection was achieved by lowering the photoluminescence of a fluorophore-labeled (QD or Alexa Fluor 488) antibody. The antibody-conjugated QD (EAb-QD) functions as a donor, while (AuNP-CBC) plays the role of acceptor in this setting. Bacterial interaction leads to a decrease in photoluminescence as shown in Figure 15. The author suggested that the decrease in distance decreases between the fluorophore and quencher plays an important role in promoting the FRET phenomena for bacterial detection. As *E. coli* is captured by AuNP-CBC/EAb-QD or AuNP-CBC/EAb-AF, it causes conformational rearrangement in the antibody structure.

This promotes FRET energy transfer, resulting in the quenching of fluorophore and a significant decrease in the photoluminescence. A detection limit of 50 CFU mL^−1^ has been observed in a standard buffer using this approach.

This work highlights the significance of facile bacterial detection for food safety applications, based on a simplified plasmonic-based structure. In another finding, Zhang et al. demonstrated the fabrication of a FRET biosensor for bacterial detection using aptamer-modified quantum dots (Aptamer-QDs) acting as a donor, while lectin concanavalin A (Con A)-modified gold nanoparticles (Con A-AuNPs) served as an acceptor [64]. The FRET played a vital role in detecting *Escherichia coli* (*E. coli*) achieving an LOD of 45 CFU/mL. The developed system also demonstrated good function in detecting *E. coli* from milk, juices, etc. Ren et al. demonstrated a paper-based FRET biosensor integrated with smart phone for detecting Vibrio parahaemolyticus in seafood [65]. It was achieved by the integration of carbon dots (CDs) with amino-modified single stranded DNA. The fabricated sensor achieved a detection limit of 8.9 CFU/mill. This study highlights a simplified approach for the fabrication of FRET biosensors for bacterial detection.

**Figure 16:**
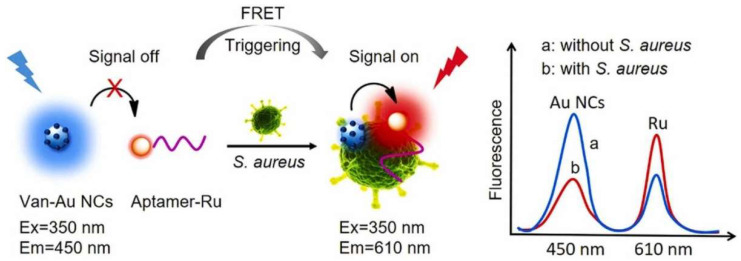
Schematic diagram for fabrication of Van-Au NCs and aptamer-Ru-based FRET sensor for the detection of *S. aureus*. Reproduced with permission from reference [66].

The applications for FRET biosensors have been extended for clinical applications. For instance, a Au NCs donor- and Ru(bpy)_3_^2+^ acceptor-based FRET biosensor has been designed for the detection of *Staphylococcus aureus* (*S. aureus*) in human serum. The sensor construct was composed of a vancomycin hydrochloride (Van) anchor, gold nanocluster (Au NCs), and an aptamer as shown in Figure 16. The fluorescence intensity decreased for the case of Au and increased for Ru(bpy)_3_^2+^, as reflected by the increasing *S. aureus* concentration, which confirms the execution of the FRET phenomena. The designed sensor achieved an LOD of 1.0 CFU/mL [66]. Initially, the system is in a “signal off” state due to FRET quenching between Van-Au NCs and Aptamer-Ru, where the energy transfer is blocked. Upon the addition of *S. aureus*, the aptamer binds to the bacteria, triggering a conformational change that disrupts the FRET, turning the system to a “signal on” state, with enhanced fluorescence at 610 nm. This is confirmed by fluorescence measurements showing a low signal at 450 nm without *S. aureus* and a strong peak at 610 nm with *S. aureus*, indicating successful detection. The simple approach demonstrated in this work is useful for early diagnosis of bacterial infections to improve public health. Recently, genetically encoded FRET biosensors have been developed by exploiting scarlet-derived green fluorescent proteins. These proteins have the advantages of having low toxicity in nature and higher spectral separations. The designed sensor demonstrated the effective detection of protease activity, Ca^2+^, and K^+^ [67]. The above findings reflect the capabilities of FRET biosensors for the accurate and sensitive detection of bacteria. However, aside from the significant development in this direction, there are certain existing challenges such as the interference of other bacteria and the need to further enhance the sensitivity of sensors to ensure precise and efficient bacterial detection. Moreover, tailoring FRET efficiency holds strong potential to significantly boost the detection capabilities of biosensors for bacterial detections.

#### 2.3.2. Detection of Viral Pathogens

Recently, viral infections causing outbreaks have emerged as a threat to global health. In particular, the recent pandemic of COVID-19 seriously impacted health and economic activities. Aside from adopting counter measures such as vaccine development, it is equally important to develop rapid detections tools for the identification of viruses. Current approaches of viral identifications such as those based on PCR are considered as well-established routes; however, they may have demerits including sophisticated equipment and a high cost for diagnosis. In addition, these are time-consuming to obtain the required results. Therefore, the development of a rapid test for virus identification at an affordable price could be advantageous to deal with such shortcomings. In this regard, extending the application of biosensors for the detection of various viruses holds great potential. In particular, the application of FRET biosensors for rapidly detecting viruses has emerged as an important diagnostic tool. They offer the benefits of early-stage detection of infection, having high sensitivity with rapid detection. Such unique features offered by FRET biosensors could be helpful in preventing viral pandemics and improving the global health [68].

**Figure 17:**
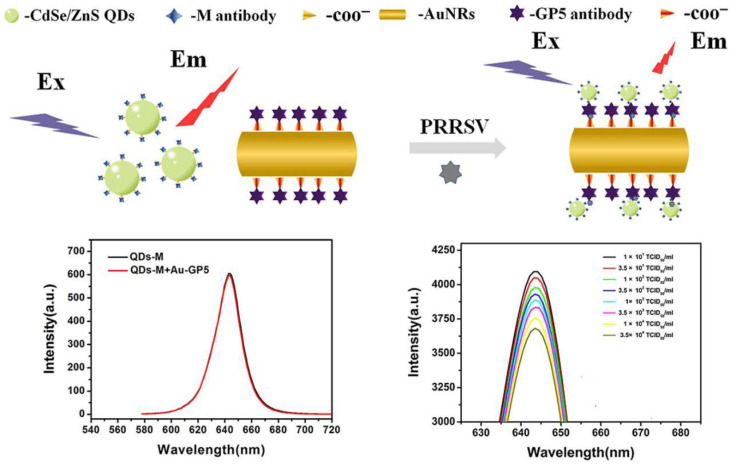
Schematic diagram of the conjugation of M and GP5 antibodies with CdSe/ZnS QDs and Au NR. Fluorescence spectra of CdSe/ZnS-M QDs and AuNRs-GP5 bioprobes in the absence of PRRSV and with different concentrations of PRRSV. Reproduced with permission from reference [70].

FRET biosensors have been developed to detect diverse viruses, including Rotavirus, Ebola, Influenza HIV, and Hepatitis C. For such sensors, the critical factors governing the sensing ability of viruses are dependent upon the FRET efficiency. The sensitivity and detection limit can be tuned by tuning the optical properties of FRET components to realize an efficient platform for biosensing [69]. The most recent strategies adopted for viral detection using FRET biosensors have been outlined as follows. The porcine reproductive and respiratory syndrome virus (PRRSV) found in animal causes infectious disease. Li et al. [70] designed a CdSe/ZnS quantum dots (QD) and Au nanorods-based FRET biosensor for the detection of PRRSV. CdSe/ZnS QDs were integrated with antibodies M and antigen-GP5. A decrease in the fluorescence intensity of CdSe/ZnS QDs was observed, based on the concentration of the antigen, driven by the FRET energy transfer. The formation scheme of a biosensor and conjugation with antibodies is presented in Figure 17. In this design, CdSe/ZnS QDs and AuNRs acted as the donor and acceptor, respectively. Incorporating GP5 and M resulted in a decrease in the distance between CdSe/ZnS QDs and AuNRs. It resulted in the occurrence of FRET, which resulted in the fluorescence quenching of QDs by AuNRs. The concentration of the antigen was detected by measuring the change in the florescence intensity. It also shows that, when no antigen was present, no quenching in fluorescence was noticed. In contrast, a gradual decrease in fluorescence was noticed when the concentration of PRRSV concentration was raised gradually. This design has resulted in an LOD of 0.55 TCID50/mL. This finding highlights the potential of FRET-based biosensors for detecting viral infections in animals [70].

**Figure 18:**
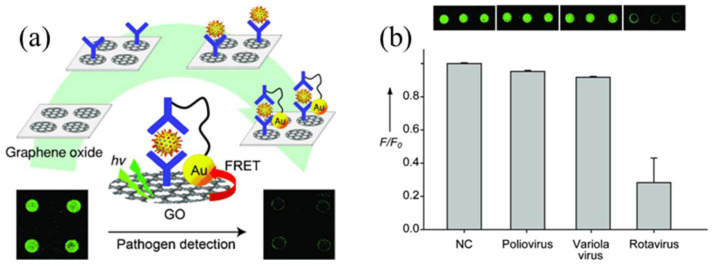
(**a**) Schematic diagram for fabrication of a GO-based FRET biosensor to detect viral pathogens and (**b**) the fluorescence quenching effect of a GO-based immuno-biosensor for various viruses. Adopted from reference [72].

Santos et al. [71] demonstrated a significant advancement in a QD-based FRET biosensor for imaging analysis. They demonstrated a stable platform for highly sensitive imaging for in vivo analysis in zebrafish. The signal-to-noise ratio was greatly enhanced with a minimized interference of autofluorescence [71]. Two-dimensional carbon materials such as graphene oxide (GO) have emerged as potential candidates for fabricating FRET-based biosensors for virus detection. The two-dimensional structure, large surface area, and tunable optical features make them attractive for fabricating biosensors for detecting various pathogens. Jung et al. investigated the role of GO in detecting rotavirus; in their work, they combined GO with Au NP for PL quenching, driven by the FRET [72]. The fabrication of the GO-based sensor is shown in Figure 18. In this work, carbodiimide was utilized for immobilizing antibodies on GO. Pathogen detection is linked to a decrease in the fluorescence emission, as shown in Figure 18. The specificity test performed by the designed sensor reflected that other viruses such as poliovirus and variola virus showed quite lower quenching in fluorescence, whereas rotavirus showed a significant reduction in fluorescence emission.

**Figure 19:**
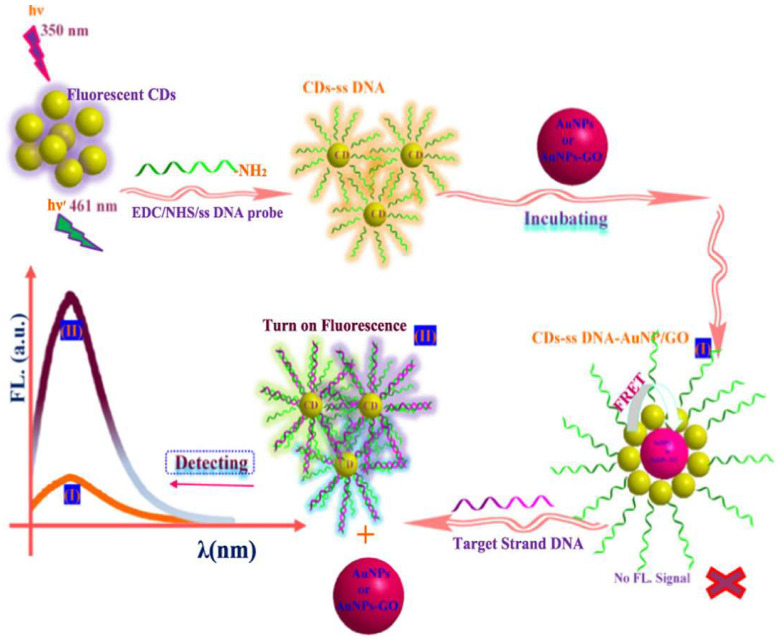
The mechanism for the FRET-based detection system. Reproduced with permission from reference [73].

It has been confirmed that the designed biosensor can be selectively applied for the detection of rotavirus. The designed sensor resulted in an LOD of 10^5^ pfu mL^−1^. Qaddare et al. demonstrated the use of carbon dots (CDs) obtained from histidine as a fluorophore for fabricating a FRET-based biosensor for HIV-1 gene detection [73]. The LOD of the designed sensing platform was found to be 15 fM. In this work, CDs served as donors, whereas AuNPs and AuNPs/GO played the role of quenching the fluorescence, which was related to detecting HIV DNA. The fabrication of the FRET biosensor is highlighted in Figure 19. Furthermore, the experiment involved a fluorescence-based detection system, where fluorescent CdS nanoparticles were conjugated with single-stranded DNA (ssDNA) probes using EDC/NHS chemistry, emitting at 461 nm when excited at 350 nm. These CdS-ssDNA conjugates were incubated with AuNPs or AuNPs-GO, initially quenching the fluorescence due to FRET, resulting in no detectable signal. Upon addition of the target strand DNA, the CdS-ssDNA detached from the AuNPs or AuNPs-GO, turning on the fluorescence, which was detected as a peak of 461 nm, indicating the presence of the target DNA, while the absence of the target DNA maintains the quenched no-fluorescence state.

**Figure 20:**
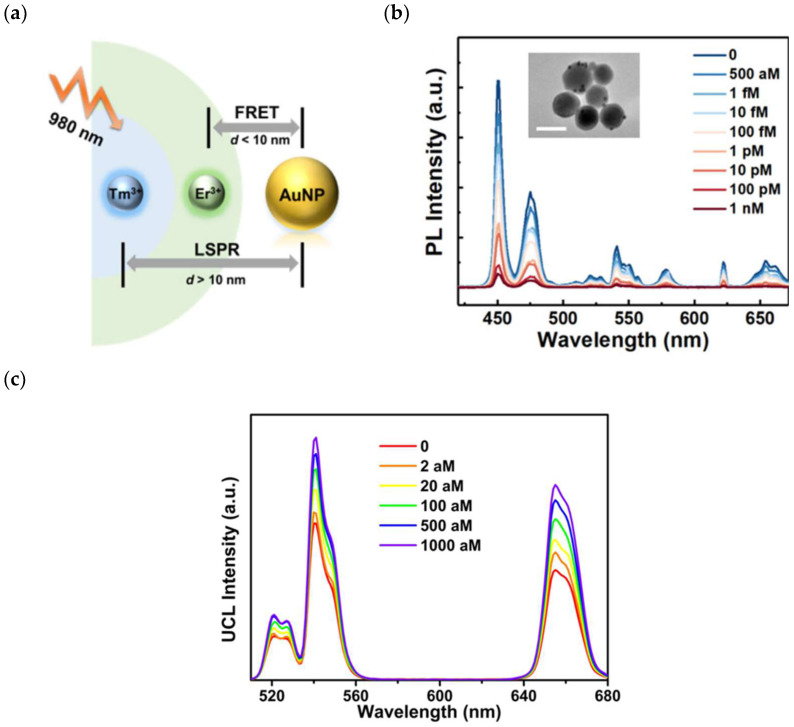
(**a**) Schematic diagram of csUCNP- and AuNP-based PE-FRET biosensor, (**b**) up-conversion spectra of csUCNP probe with various concentrations of SARS-CoV-2, with the TEM image given in inset (scale bar = 50 nm) adopted from reference [74], and (**c**) UCL spectra of csUCNP/Au-Au dimer with various concentrations of N-gene short oligos. Reproduced with permission from reference [75].

Lao et al. demonstrated the up-conversion-based FRET biosensor for the highly sensitive detection of SARS-CoV-2 RNA [74]. In this work, Tm^3+^- and Er^3+^-doped csUCNP NaGdF_4_:Yb/Tm@NaYF_4_:Yb/E up-conversion nanoparticles were prepared. The Au NP acted as the acceptor, in addition to participation in localized surface plasmon resonance (LSPR). The schematic diagram of the csUCNP- and AuNP-based PE-FRET biosensor is shown in Figure 20a. Figure 20b indicates that the quenching efficiency was enhanced as the concentration of the SARS-CoV-2 target sequence was increased. Thanks to the contribution of up-conversion and LSPR effect, the highly sensitive detection of 750 aM was achieved for SARS-CoV-2 RNA. This work demonstrates the significance of up-converting nanoparticles for designing a highly sensitive biosensor for SARS-CoV-2. Ma et al. also highlighted the potential of up-converted nanoparticles to design a FRET biosensor for the viral diagnosis of the SARS-CoV-2 N-gene. Their FRET system was composed of NaGdF_4_:Yb^3+^ and Er^3+^@NaGdF_4_ core–shell UCNPs (csUCNPs) linking with a Au–Au dimer, as shown in Figure 20c. In their work, they used Er^3+^@NaGdF_4_ core–shell UCNPs (csUCNPs) as up-conversion nanoparticles, which were integrated with a Au-Au dimer [75]. The authors claimed that FRET enabled the quenching efficiency of the Au-Au dimer to UCNPS, which resulted in up-conversion luminescence (UCL) recovery, as shown in Figure 20c.

The metal–organic framework (MOF) provides a unique platform for biosensing applications due to its porous structure and large specific surface area. These are commonly applied for designing fluorescent biosensors. In a recent work, Elgazar et al. highlighted the fabrication of a Zeolitic Imidazolate Framework-8 (ZIF-8)-derived biosensor to detect COVID-19 [76]. A P-DNA@MOF as the sensing platform was established by exploiting π–π interactions between the MOF and single single-stranded probe DNA.

**Figure 21:**
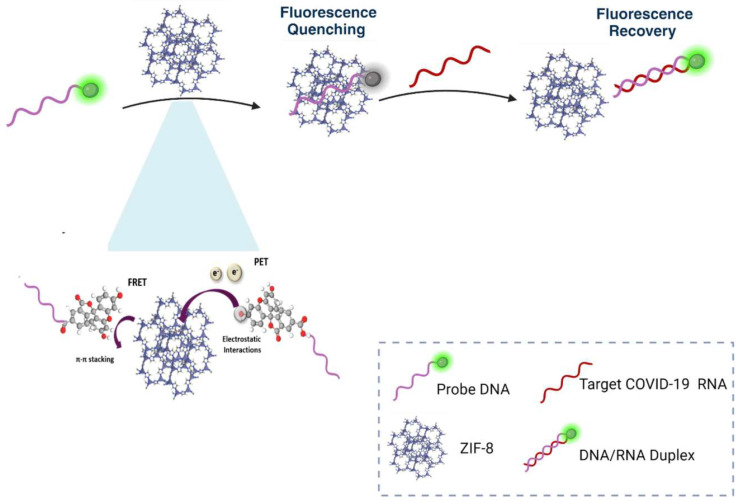
Detection mechanism of ZIF-8-based fluorescent biosensor for the target COVID-19 RNA. Reproduced with permission from reference [76].

It achieved the highly sensitive detection of COVID-19 RNA with an LOD of 6.24 pM. However, it was observed that FRET contributed relatively less to quenching P-DNA, while photoinduced electron transfer (PET) played a dominant role in the quenching process. The principle of the MOF-based FRET biosensor is given in Figure 21. This experiment utilized a fluorescence-based detection system involving a ZIF-8 (zeolitic imidazolate framework-8) nanostructure conjugated with a probe DNA labeled with a fluorophore, which initially exhibited fluorescence quenching due to FRET and PET interactions, including π-π stacking and electrostatic interactions. Upon the addition of target COVID-19 RNA, the probe DNA hybridized with the RNA to form a DNA/RNA duplex, displacing it from the ZIF-8 surface, which disrupted the quenching effect and allowed fluorescence recovery, enabling the detection of the target RNA through the restored emission signal.

#### 2.3.3. Detection of Fungal Pathogens

Invasive fungal infections (IFI) are responsible for causing fatal illness. Yang et al. developed a conjugated polymer-based fluorescence resonance energy transfer (CCP-FRET) for detecting multiple types of fungal pathogens. FRET-based sensors have shown higher sensitivity compared to the PCR method, reflecting a significant improvement in pathogen detection [77].

Various other approaches such as electrochemical methods have been applied for detecting fungal pathogens [78,79]. The application of FRET technology holds unique advantages in term of sensitive detection, higher specificity, real-time detection, and easier application, compared to other strategies. Such characteristics enable them to be a potential diagnostic tool for pathogenic detection in the food industry, clinical settings, and environmental applications. Furthermore, the integration of FRET biosensors with other analytical strategies such as microfluidic systems could further extend their application for fungal detection [80]. In addition, their emerging applications for detecting various fugal pathogens, complexity in sample composition, method validation, and development of calibration standard remain challenging. Continuous research and development are needed to overcome such challenges to expand the applications of FRET biosensors for fungal detections. FRET biosensors have immense potential in revolutionizing pathogenic fungal detection. Further advancements in this direction could substantially enhance public health.

### 2.4. Cancer Research

**Figure 22:**
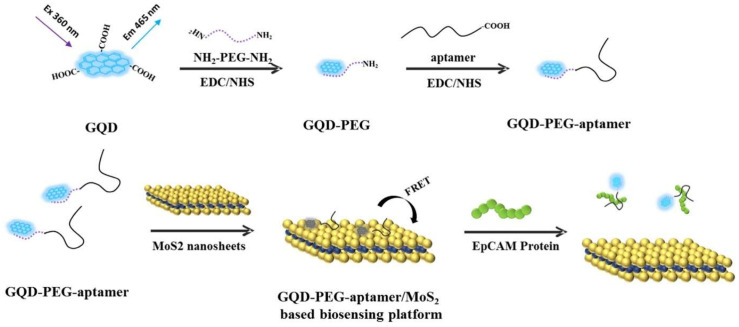
Schematic diagram for the fabrication of a GO-PEG-aptameter/MoS_2_ FRET biosensor to detect the EpCAM protein. Reproduced with permission from reference [82].

Cancer represents the abnormal growth of cells leading to health complications, one of the leading causes of deaths globally. Aside from intensive efforts toward developing cancer treatment, it is highly desirable to modernize cancer detection strategies, especially the early stage of detection, which could lower the death rate. Various strategies such as positron emission tomography (PET), X-ray computed tomography (CT), and magnetic resonance spectroscopy (MRS) have been developed for the detection of cancer [81]. FRET fluorescent-based sensors, mainly due to their easy fabrication and high sensitivity, provide unique advantage for detecting various kinds of cancer biomarkers. For instance, Shi et al. investigated the graphene quantum dots (GQDs) and molybdenum disulfide (MoS_2_) nanosheets to construct a FRET biosensor to detect the epithelial cell adhesion molecule (EpCAM) [82]. In this configuration (Figure 22), GQDs played the role of donor, and MoS_2_ served as the acceptor.

Initially, GQDs are functionalized with PEG to form GQD-PEG, followed by conjugation with an aptamer to create a GQD-PEG-aptamer, which exhibits fluorescence with excitation at 360 nm and emission at 465 nm. This aptamer-functionalized GQD is then assembled onto MoS_2_ nanosheets, forming a GQD-PEG-aptamer/MoS_2_ platform, where fluorescence is quenched due to FRET. Upon binding with the EpCAM protein, the aptamer conformation changes, disrupting the FRET and recovering the fluorescence, enabling sensitive detection of the protein.

**Figure 23:**
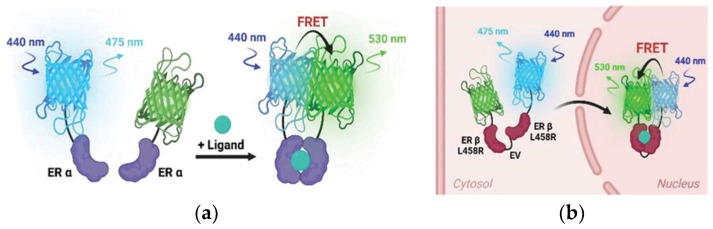
(**a**) Schematic diagram and (**b**) mode of action of the ER ββ FL-translocated FRET biosensor. Reproduced with permission from reference [60].

Li et al. [83] designed a FRET biosensor to detect the glypican-3(GPC3) antigen. In this design, GPC3 aptamer-labelled gold carbon dots (AuCDs-GPC3Apt) served as the donors, while Fe_3_O_4_/GO was the acceptor. The fluorescence intensity was recovered by the addition of GPC3, which was quantified GPC3. The sensitive detection of GPC3 facilitates the early detection of hepatocellular carcinoma (HCC). The LOD by this sensor was 3.01 ng·mL^−1^. This finding describes the use of a magnetically separable design for the efficient detection of GPC3 [83]. Han et al. used a FRET biosensor for the detection of estrogen receptors (ERs) [60]. Through optimizing linker and protein pairs, they have optimized the sensing ability to monitor the dynamics of dimer formation by distinguishing homo or hetero dimers (Figure 23). They investigated the stiffness of the extracellular matrix (ECM) affected by ER β dynamics. The authors claim that ECM stiffness can lead to ER-mediated gene expression in cancer cells.

**Figure 24:**
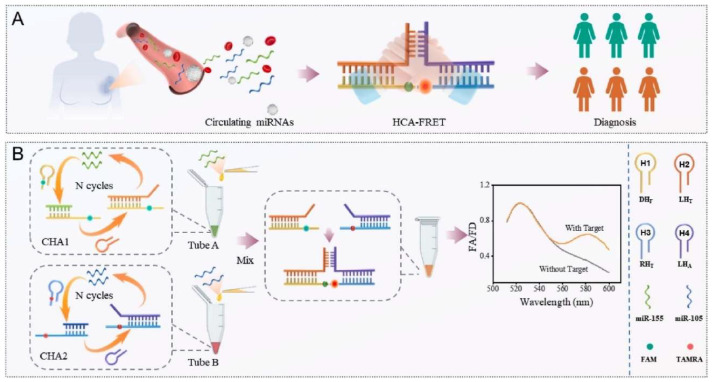
(**A**) The HCA-FRET to distinguish breast cancer patients from normal subjects by utilizing circulating miRNAs. (**B**) The design principle of the hand-in-hand catalytic hairpin assembly-based (HCA-FRET) biosensor. Reproduced with permission from reference [84].

Afshan et al. demonstrated the application of a FRET biosensor for the detection of breast cancer [84]. They demonstrated the simultaneous detection of miR-155 and miR-105 biomarkers for the diagnosis of breast cancer. The respective limits of detection for miR-155 and miR-105 were observed as 0.02 and 0.05 pM, respectively. This strategy highlights the detection of multiple biomarkers for cancer to achieve the higher accuracy for cancer detection. The schematic diagram and working principle of the designed FRET biosensor is shown in Figure 24. In this work, four hairpin probes, such as DH_F_, LH_T_, RH_T_, and LH_A_ are involved in obtaining the FRET signal. Although FRET biosensors provide distinct merits in cancer research, it remains challenging to translate them into clinical application. Certain critical aspects including complicated sensor design, possible interference in biological systems, and the lack of a calibration standard limit their applications. Besides these challenges, ongoing innovations to develop FRET technology with sensitive detection and a highly specific response continuously enhances their applications for cancer diagnosis and therapeutic applications. 

Additionally, the experiment employed a hybrid chain assembly-FRET (HCA-FRET) method to diagnose diseases by detecting circulating miRNAs from blood samples, as illustrated in Figure 24A, where miRNAs are extracted, amplified via HCA-FRET, and used for diagnostic purposes. Figure 24B details the process: two sets of hairpin DNA probes (CHA1 in Tube A and CHA2 in Tube B) undergo N cycles of hybridization chain reaction (HCR) to amplify the signal, with the resulting mixtures combined and mixed with a sample in a tube; the FRET is measured using fluorophores FAM and TAMRA, showing increased FAD (FRET acceptor/donor ratio) at a 520–580 nm wavelength with target miRNAs (e.g., miR-155, miR-105) compared to no signal without targets, enabling sensitive detection and diagnosis.

## 3. Challenges and Limitations of FRET Biosensors

Although considerable progress has been made in the field of FRET biosensors for diverse biological applications, certain challenges exist in their applications. The following limitations of in-vitro FRET assays limit their full-scale application. The fluorescence behavior of FRET biosensors is highly sensitive to environmental conditions including pH, temperature, and ionic strength. This could affect the sensitivity and response of FRET biosensors; hence, careful optimization of these conditions is needed [85,86,87]. Photobleaching of fluorophores greatly limits the durability and stability of FRET biosensors. It is desired to choose stable fluorophores of FRET biosensors to overcome this issue [88]. The key aspect in FRET biosensors, which governs the efficiency, is the distance between the donor and acceptor species. A proximity within 10 nm is expected to give better performance. Therefore, the donor–acceptor distance of a FRET should be precisely tuned. FRET biosensors are commonly applied for in vitro analysis, while their use for in vivo studies is less developed. The interference of a complicated biological environment is a potential limitation of the successful application of FRET biosensors for in vivo application. The key challenges for the in vivo application of FRET biosensors are summarized as follows. Eder et al. demonstrated the hurdles for applying FRET biosensors in Drosophila tissues, and they suggested that possible conformation of E-Cadherin or the neighboring protein affected the sensor ability [89]. Furthermore, in vivo application of a FRET biosensor faces selectivity issues, making it difficult to precisely detect interactions at molecular levels [90]. Another important aspect of using FRET biosensors is the potential toxic effect on living cells. Some of the fluorescent proteins adopted for FRET biosensors can express phototoxicity [91]. FRET emission absorbed by biological tissues limits the deeper imaging of tissues, and the penetration depth can be optimized by shifting the FRET emissions towards the NIR region [92].

## 4. Summary and Outlook of FRET Biosensors

Recent advancements in FRET biosensors have expanded their widespread usage in various directions such as healthcare, food science, and drug discovery. The main developmental aspect in such applications is based on tailoring the donor–acceptor structures for maximizing the FRET efficiency to obtain maximum outcome from their corresponding applications. Such advancements have resulted in enhancing the sensing ability, improving the specificity, and detailing the reaction mechanism of drug discovery and protein interactions in complex biological environments. The real-time monitoring of the FRET process has boosted the understanding at the molecular level and at the cellular scale. FRET biosensors work by probing the energy transfer from the donor to acceptor fluorophore. Various aspects related to engineering donors and acceptors can modulate the sensing abilities of sensors. For instance, red-shifted donor/acceptor pairs have shown reduced background noise and enhanced signal-to-noise ratios [53]. This clearly demonstrates the potential influence of fluorophore engineering for enhancing the quality of signals generated by FRET process. FRET biosensors’ capabilities to monitor real-time metabolites reflect their significance in drug discoveries and screening [48]. FRET biosensors have demonstrated higher sensing abilities than conventionally adopted PCR analysis in tracking pathogenic detection. The integration of artificial intelligence (AI) with FRET biosensors can significantly advance this field. Integration of machine learning and AI algorithms with FRET biosensors have facilitated the interpretation of fluorescence signals and the precise detection of biological molecules with improved sensitivity and accuracy. Moreover, the specificity and selectivity of FRET biosensors can be greatly enhanced by applying AI at a single molecular level, which could be helpful for the earlier detection of diseases [41].

This can lead to the development of cutting-edge technology for achieving the real-time analysis of FRET biosensors, which can revolutionize the field of biosensing technologies with the development of state-of-the-art smart devices for diversified applications ranging from food science to biological research. This can also play a vital role in drug discoveries to tackle critical illnesses, including Alzheimer’s, Parkinson’s, and dementia, through drug screening, understanding protein interactions, and tracking real-time analysis. Aside from their great potential, certain critical challenges exist in this field, which limit the wider application of FRET biosensors for various applications. FRET biosensor devices need precise calibration for real-time applications to enhance the confidence of their measurement. AI can be helpful to design novel strategies for the calculation of FRET efficiency and the quantification of charge transfer, which will enhance the reliable performance of FRET biosensors [14]. However, there could be certain challenges too, related to any bias in AI algorithms. It is important to address such challenges to obtain the maximum outcome of integrating FRET biosensors with emerging technologies. This will extend the application of FRET biosensors across wide fields.

## Data Availability

Not applicable.

## References

[1] Zadran S., Standley S., Wong K., Otiniano E., Amighi A., Baudry M. (2012). Fluorescence resonance energy transfer (FRET)-based biosensors: Visualizing cellular dynamics and bioenergetics. Appl. Microbiol. Biotechnol..

[2] Xia H., Xie K., Zou G. (2017). Advances in spiropyrans/spirooxazines and applications based on fluorescence resonance energy transfer (FRET) with fluorescent materials. Molecules.

[3] Clapp A.R., Medintz I.L., Mattoussi H. (2006). Förster resonance energy transfer investigations using quantum-dot fluorophores. ChemPhysChem.

[4] Chen G., Song F., Xiong X., Peng X. (2013). Fluorescent nanosensors based on fluorescence resonance energy transfer (FRET). Ind. Eng. Chem. Res..

[5] Wu L., Huang C., Emery B.P., Sedgwick A.C., Bull S.D., He X.-P., Tian H., Yoon J., Sessler J.L., James T.D. (2020). Förster resonance energy transfer (FRET)-based small-molecule sensors and imaging agents. Chem. Soc. Rev..

[6] Yuan L., Lin W., Zheng K., Zhu S. (2013). FRET-based small-molecule fluorescent probes: Rational design and bioimaging applications. Acc. Chem. Res..

[7] Hong S., Samson A.A.S., Song J.M. (2020). Application of fluorescence resonance energy transfer to bioprinting. TrAC Trends Anal. Chem..

[8] Yang W.C., Li S.Y., Ni S., Liu G. (2024). Advances in FRET-based biosensors from donor-acceptor design to applications. Aggregate.

[9] Fan C., Plaxco K.W., Heeger A.J. (2005). Biosensors based on binding-modulated donor–acceptor distances. TRENDS Biotechnol..

[10] Schaaf T.M., Li A., Grant B.D., Peterson K., Yuen S., Bawaskar P., Kleinboehl E., Li J., Thomas D.D., Gillispie G.D. (2018). Red-shifted FRET biosensors for high-throughput fluorescence lifetime screening. Biosensors.

[11] Hirata E., Kiyokawa E. (2016). Future perspective of single-molecule FRET biosensors and intravital FRET microscopy. Biophys. J..

[12] Aoki K., Komatsu N., Hirata E., Kamioka Y., Matsuda M. (2012). Stable expression of FRET biosensors: A new light in cancer research. Cancer Sci..

[13] Tao W., Rubart M., Ryan J., Xiao X., Qiao C., Hato T., Davidson M.W., Dunn K.W., Day R.N. (2015). A practical method for monitoring FRET-based biosensors in living animals using two-photon microscopy. Am. J. Physiol.-Cell Physiol..

[14] Wu J.-W., Yang J.-M., Chen C.-C., Au G., Wang S., Chern G.-W., Huang C.-H. (2024). Calibration of FRET-based biosensors using multiplexed biosensor barcoding. bioRxiv.

[15] Wu W., Bazan G.C., Liu B. (2017). Conjugated-polymer-amplified sensing, imaging, and therapy. Chem.

[16] Pini F., Francés-Soriano L., Andrigo V., Natile M.M., Hildebrandt N. (2023). Optimizing upconversion nanoparticles for FRET biosensing. ACS Nano.

[17] Kong X., Nir E., Hamadani K., Weiss S. (2007). Photobleaching pathways in single-molecule FRET experiments. J. Am. Chem. Soc..

[18] Levitt J.A., Poland S.P., Krstajic N., Pfisterer K., Erdogan A., Barber P.R., Parsons M., Henderson R.K., Ameer-Beg S.M. (2020). Quantitative real-time imaging of intracellular FRET biosensor dynamics using rapid multi-beam confocal FLIM. Sci. Rep..

[19] Tao Y., Chen L., Pan M., Zhu F., Zhu D. (2021). Tailored biosensors for drug screening, efficacy assessment, and toxicity evaluation. ACS Sens..

[20] Zhang W., Li W., Song Y., Xu Q., Xu H. (2024). Bacterial detection based on Förster resonance energy transfer. Biosens. Bioelectron..

[21] Ouyang N., Hong L., Zhou Y., Zhang J., Shafi S., Pan J., Zhao R., Yang Y., Hou W. (2022). Application of fluorescent nano-biosensor for the detection of cancer bio-macromolecular markers. Polym. Test..

[22] Kaur A., Kaur P., Ahuja S. (2020). Förster resonance energy transfer (FRET) and applications thereof. Anal. Methods.

[23] Bai S., Zhang P., Beratan D.N. (2020). Predicting dexter energy transfer interactions from molecular orbital overlaps. J. Phys. Chem. C.

[24] Baibakov M., Patra S., Claude J.-B., Moreau A., Lumeau J., Wenger J. (2019). Extending single-molecule Forster resonance energy transfer (FRET) Range beyond 10 nanometers in zero-mode waveguides. ACS Nano.

[25] Zhang R., Zhong H., Yang K., Pan K., Zhao B., Deng J. (2025). Energy transfer for constructing circularly polarized luminescence materials: Recent progress and future prospects. Adv. Funct. Mater..

[26] Rowland C.E., Brown C.W., Medintz I.L., Delehanty J.B. (2015). Intracellular FRET-based probes: A review. Methods Appl. Fluoresc..

[27] Jin Z., Dridi N., Palui G., Palomo V., Jokerst J.V., Dawson P.E., Sang Q.-X.A., Mattoussi H. (2023). Quantum Dot–Peptide Conjugates as Energy Transfer Probes for Sensing the Proteolytic Activity of Matrix Metalloproteinase-14. Anal. Chem..

[28] Majumder S., Deb S., Hussain S., Dey D., Bhattacharjee D., Alodhayb A.N., Hussain S., Hussain S.A. (2025). Spectroscopic investigation of two xanthane dyes and design of a FRET based pesticide sensor. Sci. Rep..

[29] Liu S., Akram W., Ye F., Jin J., Niu F., Ahmed S., Ouyang Z., Dong S.C., Li G. (2025). Förster Resonance Energy Transfer in Metal Halide Perovskite: Current Status and Future Prospects. ChemistryOpen.

[30] Stryer L. (1978). Fluorescence energy transfer as a spectroscopic ruler. Annu. Rev. Biochem..

[31] Roy R., Hohng S., Ha T. (2008). A practical guide to single-molecule FRET. Nat. Methods.

[32] Liu S., Liu J., Foote A., Ogasawara H., Al Abdullatif S., Batista V.S., Salaita K. (2025). Digital and Tunable Genetically Encoded Tension Sensors Based on Engineered Coiled-Coils. Angew. Chem. Int. Ed. Engl..

[33] Zou Y., Long S., Xiong T., Zhao X., Sun W., Du J., Fan J., Peng X. (2021). Single-Molecule Förster Resonance Energy Transfer-Based Photosensitizer for Synergistic Photodynamic/Photothermal Therapy. ACS Cent. Sci..

[34] Marras A.E., Shi Z., Lindell M.G., Patton R.A., Huang C.-M., Zhou L., Su H.-J., Arya G., Castro C.E. (2018). Cation-Activated Avidity for Rapid Reconfiguration of DNA Nanodevices. ACS Nano.

[35] Talbott J.M., Wills R., Shirke R., Hassanein L., Weinshenker D., Raj M. (2025). Spatiotemporal Imaging of Catechol Aldehydes in Neural Tissue. JACS Au.

[36] Mavileti S.K., Bila G., Utka V., Bilyy R., Bila E., Butoi E., Gupta S., Balyan P., Kato T., Bilyy R. (2025). Squaraine-Peptide Conjugates as Efficient Reporters of Neutrophil Extracellular Traps-Mediated Chronic Inflammation. ACS Appl. Mater. Interfaces.

[37] Lee J., Lai S., Yang S., Zhao S., Blanco F.A., Lyons A.C., Merino-Urteaga R., Ahrens J.F., Nguyen N.A., Liu H. (2025). Bright and photostable yellow fluorescent proteins for extended imaging. Nat. Commun..

[38] Kagan T., Gabay M., Meenakshisundaram A., Levi Y., Eid S., Malchenko N., Maman M., Nitzan A., Ravotto L., Zaidel-Bar R. (2025). Genetically encoded biosensor for fluorescence lifetime imaging of PTEN dynamics in the intact brain. Nat. Methods.

[39] Soleja N., Mohsin M. (2024). Exploring the landscape of FRET-based molecular sensors: Design strategies and recent advances in emerging applications. Biotechnol. Adv..

[40] Vu C.Q., Arai S. (2023). Quantitative Imaging of Genetically Encoded Fluorescence Lifetime Biosensors. Biosensors.

[41] Verma A.K., Noumani A., Yadav A.K., Solanki P.R. (2023). FRET Based Biosensor: Principle Applications Recent Advances and Challenges. Diagnostics.

[42] Chen H., Ding F., Zhou Z., He X., Shen J. (2020). FRET-based sensor for visualizing pH variation with colorimetric/ratiometric strategy and application for bioimaging in living cells, bacteria and zebrafish. Analyst.

[43] Yang D., Sun R., Sun H., Li Q., Zhang H., Zhang X., Shi L., Yao L., Tang Y. (2025). A FRET biosensor constructed using pH sensitive G-quadruplex DNA for detecting mitochondrial autophagy. Talanta.

[44] Svensson B., Nitu F.R., Rebbeck R.T., McGurran L.M., Oda T., Thomas D.D., Bers D.M., Cornea R.L. (2023). Molecular Mechanism of a FRET Biosensor for the Cardiac Ryanodine Receptor Pathologically Leaky State. Int. J. Mol. Sci..

[45] Ouyang M., Xing Y., Zhang S., Li L., Pan Y., Deng L. (2024). Development of FRET Biosensor to Characterize CSK Subcellular Regulation. Biosensors.

[46] Ouyang M., Sun J., Chien S., Wang Y. (2008). Determination of hierarchical relationship of Src and Rac at subcellular locations with FRET biosensors. Proc. Natl. Acad. Sci. USA.

[47] Kelly T., Yang X. (2024). Application of Fluorescence- and Bioluminescence-Based Biosensors in Cancer Drug Discovery. Biosensors.

[48] Chang H., Clemens S., Gao P., Li Q., Zhao H., Wang L., Zhang J., Zhou P., Johnsson K., Wang L. (2024). Fluorogenic rhodamine-based chemigenetic biosensor for monitoring cellular NADPH dynamics. J. Am. Chem. Soc..

[49] Roopnarine O., Yuen S.L., Thompson A.R., Roelike L.N., Rebbeck R.T., Bidwell P.A., Aldrich C.C., Cornea R.L., Thomas D.D. (2023). Fluorescence lifetime FRET assay for live-cell high-throughput screening of the cardiac SERCA pump yields multiple classes of small-molecule allosteric modulators. Sci. Rep..

[50] Muretta J.M., Rajasekaran D., Blat Y., Little S., Myers M., Nair C., Burdekin B., Yuen S.L., Jimenez N., Guhathakurta P. (2023). HTS driven by fluorescence lifetime detection of FRET identifies activators and inhibitors of cardiac myosin. SLAS Discov..

[51] Schultz R.L., Yuen S.L., Rebbeck R.T., Svensson B., Roopnarine O., Thomas D.D. (2024). Live-cell FRET biosensors for high-throughput screening targeting the SERCA2A-DWORF complex. Biophys. J..

[52] Yu H., Feng R., Chen F., Wu Z., Li D., Qiu X. (2024). Rapid FRET Assay for the Early Detection of Alpha-Synuclein Aggregation in Parkinson’s Disease. ACS Chem. Neurosci..

[53] Braun A.R., Kochen N.N., Yuen S.L., Liao E.E., Cornea R.L., Thomas D.D., Sachs J.N. (2023). Advancements in a FRET Biosensor for Live-Cell Fluorescence-Lifetime High-Throughput Screening of Alpha-Synuclein. ASN Neuro.

[54] Wu G., Xiang Y., Liu H., Hu C., Li Y., Feng J., Li Y. (2025). Virtual Screening Combined with Phase Transition-FRET for Discovery of Small-Molecule FcRn Antagonists. Anal. Chem..

[55] Funato Y., Mimura M., Nunomura K., Lin B., Fujii S., Haruta J., Miki H. (2024). Development of a high-throughput screening system targeting the protein-protein interactions between PRL and CNNM. Sci. Rep..

[56] Xing F., Ai N., Huang S., Jiang C., Mughal M.J., Ge W., Wang G., Deng C.-X. (2022). An In Vivo Fluorescence Resonance Energy Transfer-Based Imaging Platform for Targeted Drug Discovery and Cancer Therapy. Front. Bioeng. Biotechnol..

[57] Gu M., Zhang H., Liu Y., Li X., Lv M., Zhao J., Zhang J. (2024). Accurate and highly sensitive detection of Alzheimer’s disease-related extracellular vesicles via förster resonance energy transfer. Anal. Chim. Acta.

[58] Nathan Kochen N., Murray M., Vunnam N., Liao E.E., Chen L., Braun A.R., Sachs J.N. (2024). Fluorescence lifetime-based FRET biosensors for monitoring N-terminal domain interactions of TDP-43 in living cells: A novel resource for ALS and FTD drug discovery. bioRxiv.

[59] Kim Y., Kim S., Heo K., Lee S. (2024). Single-molecule FRET–based approach for protein-targeted drug discovery. Mol. Cells.

[60] Han K., Suh J.S., Choi G., Jang Y.K., Ahn S., Lee Y., Kim T.J. (2025). Novel FRET-Based Biosensors for Real-Time Monitoring of Estrogen Receptor Dimerization and Translocation Dynamics in Living Cells. Adv. Sci..

[61] Wijesinghe K.M., Sabbih G., Algama C.H., Syed R., Danquah M.K., Dhakal S. (2023). FRET-Based Single-Molecule Detection of Pathogen Protein IsdA Using Computationally Selected Aptamers. Anal. Chem..

[62] Zhang X., Xu J., Yan C., Yao L., Shang H., Chen W. (2021). A Short- and Long-Range Fluorescence Resonance Energy Transfer-Cofunctionalized Fluorescence Quenching Collapsar Probe Regulates Amplified and Accelerated Detection of Salmonella. J. Agric. Food Chem..

[63] Heli B., Ajji A. (2020). Toward a nanopaper-based and solid phase immunoassay using FRET for the rapid detection of bacteria. Sci. Rep..

[64] Zhang Y., Liu Y., Yang Y., Li L., Tao X., Song E. (2023). Rapid detection of pathogenic bacteria based on a universal dual-recognition FRET sensing system constructed with aptamer-quantum dots and lectin-gold nanoparticles. Chin. Chem. Lett..

[65] Ren Y., Cao L., Zhang X., Jiao R., Ou D., Wang Y., Zhang D., Shen Y., Ling N., Ye Y. (2023). A novel fluorescence resonance energy transfer (FRET)-based paper sensor with smartphone for quantitative detection of Vibrio parahaemolyticus. Food Control.

[66] Feng Q., Xu H., Li Z., Chen C., Miao X. (2022). A novel “signal off–triggered on” Förster resonance energy transfer biosensor for the ratiometric detection of pathogenic bacteria. Sens. Actuators B Chem..

[67] Gohil K., Wu S.-Y., Takahashi-Yamashiro K., Shen Y., Campbell R.E. (2023). Biosensor Optimization Using a Förster Resonance Energy Transfer Pair Based on mScarlet Red Fluorescent Protein and an mScarlet-Derived Green Fluorescent Protein. ACS Sens..

[68] Patel S.K., Surve J., Parmar J., Ahmed K., Bui F.M., Al-Zahrani F.A. (2023). Recent Advances in Biosensors for Detection of COVID-19 and Other Viruses. IEEE Rev. Biomed. Eng..

[69] Salama A.M., Yasin G., Zourob M., Lu J. (2022). Fluorescent Biosensors for the Detection of Viruses Using Graphene and Two-Dimensional Carbon Nanomaterials. Biosensors.

[70] Li X., Li J., Yang W., Han D., Yao N., Zhao H., Chu X., Liang X., Bi C., Wang C. (2022). Fluorescent immunosensor based on fluorescence resonance energy transfer between CdSe/ZnS quantum dots and Au nanorods for PRRSV detection. Prog. Nat. Sci. Mater. Int..

[71] Cardoso Dos Santos M., Colin I., Ribeiro Dos Santos G., Susumu K., Demarque M., Medintz I.L., Hildebrandt N. (2020). Time-Gated FRET Nanoprobes for Autofluorescence-Free Long-Term In Vivo Imaging of Developing Zebrafish. Adv. Mater..

[72] Jung J.H., Cheon D.S., Liu F., Lee K.B., Seo T.S. (2010). A graphene oxide based immuno-biosensor for pathogen detection. Angew. Chem. Int. Ed. Engl..

[73] Qaddare S.H., Salimi A. (2017). Amplified fluorescent sensing of DNA using luminescent carbon dots and AuNPs/GO as a sensing platform: A novel coupling of FRET and DNA hybridization for homogeneous HIV-1 gene detection at femtomolar level. Biosens. Bioelectron..

[74] Lao X., Liu Y., Li L., Song M., Ma Y., Yang M., Chen G., Hao J. (2024). Plasmon-enhanced FRET biosensor based on Tm^3+^/Er^3+^ co-doped core-shell upconversion nanoparticles for ultrasensitive virus detection. Aggregate.

[75] Ma Y., Song M., Li L., Lao X., Liu Y., Wong M.-c., Yang M., Chen H., Hao J. (2024). Attomolar-level detection of respiratory virus long-chain oligonucleotides based on FRET biosensor with upconversion nanoparticles and Au–Au dimer. Biosens. Bioelectron..

[76] Elgazar A., Sabouni R., Ghommem M., Majdalawieh A.F. (2024). Novel metal–organic framework biosensing platform for detection of COVID-19 RNA. Sci. Rep..

[77] Yang Q., He B., Chen C., Wang H., Li W., Xue X., Qiu T., Hao X., Lv F., Wang S. (2021). A Rapid, Visible, and Highly Sensitive Method for Recognizing and Distinguishing Invasive Fungal Infections via CCP-FRET Technology. ACS Infect. Dis..

[78] Rana M., Yilmaz T., Cohen S., Beyhan S., Argun A.A. (2023). A novel biosensor for ultrasensitive detection of fungal genes. Biosens. Bioelectron..

[79] Zhang X., Guo B., Wang Y., Hu L., Yang N., Mao H. (2022). A Detection Method for Crop Fungal Spores Based on Microfluidic Separation Enrichment and AC Impedance Characteristics. J. Fungi.

[80] Shi H., Wang Y., Zhang Z., Yu S., Huang X., Pan D., Wang Z., Huang Q.-A., Zhu Z. (2023). Recent advances of integrated microfluidic systems for fungal and bacterial analysis. TrAC Trends Anal. Chem..

[81] Pulumati A., Pulumati A., Dwarakanath B.S., Verma A., Papineni R.V.L. (2023). Technological advancements in cancer diagnostics: Improvements and limitations. Cancer Rep..

[82] Shi J., Lyu J., Tian F., Yang M. (2017). A fluorescence turn-on biosensor based on graphene quantum dots (GQDs) and molybdenum disulfide (MoS2) nanosheets for epithelial cell adhesion molecule (EpCAM) detection. Biosens. Bioelectron..

[83] Li G., Chen W., Mi D., Wang B., Li H., Wu G., Ding P., Liang J., Zhou Z. (2022). A highly sensitive strategy for glypican-3 detection based on aptamer/gold carbon dots/magnetic graphene oxide nanosheets as fluorescent biosensor. Anal. Bioanal. Chem..

[84] Afshan N., Cheng T., Yu J., Jiao K., Li L., Jiao J., Jiao J. (2025). Hand in hand catalytic hairpin assembly-based FÖrster resonance energy transfer biosensor for simultaneous detection of multiple MicroRNAs from breast cancer. Anal. Chim. Acta.

[85] Rennick J.J., Nowell C.J., Pouton C.W., Johnston A.P.R. (2022). Resolving subcellular pH with a quantitative fluorescent lifetime biosensor. Nat. Commun..

[86] Rana D.K., Bhattacharya S.C. (2022). Implication toward a simple strategy to generate pH tunable FRET-based biosensing. Spectrochim. Acta Part. A Mol. Biomol. Spectrosc..

[87] Leavesley S.J., Rich T.C. (2016). Overcoming limitations of FRET measurements. Cytom. A.

[88] Adhikari D.P., Stoneman M.R., Raicu V. (2025). Impact of photobleaching of fluorescent proteins on FRET measurements under two-photon excitation. Spectrochim. Acta Part. A Mol. Biomol. Spectrosc..

[89] Eder D., Basler K., Aegerter C.M. (2017). Challenging FRET-based E-Cadherin force measurements in Drosophila. Sci. Rep..

[90] Liu L., He F., Yu Y., Wang Y. (2020). Application of FRET Biosensors in Mechanobiology and Mechanopharmacological Screening. Front. Bioeng. Biotechnol..

[91] Günther E., Klauß A., Toro-Nahuelpan M., Schüler D., Hille C., Faivre D. (2019). The in vivo mechanics of the magnetotactic backbone as revealed by correlative FLIM-FRET and STED microscopy. Sci. Rep..

[92] Smith J.T., Sinsuebphon N., Rudkouskaya A., Michalet X., Intes X., Barroso M. (2023). In vivo quantitative FRET small animal imaging: Intensity versus lifetime-based FRET. Biophys. Rep..

